# Interaction of Indirect and Hyperdirect Pathways on Synchrony and Tremor-Related Oscillation in the Basal Ganglia

**DOI:** 10.1155/2021/6640105

**Published:** 2021-03-13

**Authors:** Xia Shi, Danwen Du, Yuan Wang

**Affiliations:** ^1^School of Science, Beijing University of Posts and Telecommunications, Beijing 100876, China; ^2^State Key Laboratory of Networking and Switching Technology, Beijing University of Posts and Telecommunications, Beijing 100876, China

## Abstract

Low-frequency oscillatory activity (3-9 Hz) and increased synchrony in the basal ganglia (BG) are recognized to be crucial for Parkinsonian tremor. However, the dynamical mechanism underlying the tremor-related oscillations still remains unknown. In this paper, the roles of the indirect and hyperdirect pathways on synchronization and tremor-related oscillations are considered based on a modified Hodgkin-Huxley model. Firstly, the effects of indirect and hyperdirect pathways are analysed individually, which show that increased striatal activity to the globus pallidus external (GPe) or strong cortical gamma input to the subthalamic nucleus (STN) is sufficient to promote synchrony and tremor-related oscillations in the BG network. Then, the mutual effects of both pathways are analysed by adjusting the related currents simultaneously. Our results suggest that synchrony and tremor-related oscillations would be strengthened if the current of these two paths are in relative imbalance. And the network tends to be less synchronized and less tremulous when the frequency of cortical input is in the theta band. These findings may provide novel treatments in the cortex and striatum to alleviate symptoms of tremor in Parkinson's disease.

## 1. Introduction

Parkinson's disease (PD) is a neurodegenetative disorder resulting from a loss of dopaminergic neurons, which leads to alterations of neural activity in the basal ganglia (BG), thalamus (TH), and cortex [1–3]. The BG is the main pathologic area of PD, which is known to be implicated in choice selection, timing, working memory, and motor sequencing [4–6]. Parkinsonian tremor is a well-recognized cardinal symptom affecting ~70% of PD patients, which is primarily a rest tremor with the frequency in 3-9 Hz range. “Tremor-related activity” is defined as neural activity at a frequency in the range of Parkinsonian tremor [7, 8]. Intracranial recordings of local field potentials (LFPs) and electromyography (EMG) signals recorded synchronised tremor-related activity in the subthalamic nucleus (STN) [9–11], TH [12], and the internal globus pallidus (GPi) [13]. Tremor-related activity also has been detected directly from the cortical surface as well as by electroencephalogram (EEG) and EMG [14–17]. And the pathophysiology of tremor is believed to be distinct from that of rigidity and akinesia in imaging studies [18].

A few controversial hypotheses of tremor generation have been proposed previously. One of them was STN and globus pallidus external (GPe) pacemaker hypothesis based on an experiment [19], which showed that STN and GPe interacted by their mutual connections to exhibit low frequency oscillation (0.4-1.8 Hz). Some argued that the frequency of these oscillations was below that of tremor [20]. Others suggested that the BG may be a tremor-generating oscillator on its own [21]. However, this is also debatable since lesions outside the BG (such as lesions in TH [12] and cortex [15, 17]) can suppress Parkinsonian tremor. Thus, the mechanism underlying Parkinsonian tremor has not been clearly understood.

Importantly, electrophysiological study [22] has reported increased striatal input to GPe from the indirect pathway after dopamine depletion. Recently, experimental studies [23–25] have reported that the striatum can play a key role in generating and controlling oscillations in the BG. In addition, clinical research [26] clearly demonstrated a cortical correlation with Parkinsonian tremor. And a neuroimage study [27] showed increased functional connectivity between STN and cortical areas mediated via the hyperdirect pathway in patients with tremor. In animal PD models, the hyperdirect pathway is overactive in Parkinsonian state [15]. Thus, it is possible that the mutual effect of cortical activity transmitted to the BG in a fast hyperdirect loop and the activity in the striatum from the indirect pathway is the cause of the PD tremor symptoms. However, the role of these two paths in the development of Parkinsonian tremor was rarely concerned. Although computational studies [28–33] have explored the origins of excessive oscillations in the BG, the oscillatory activities in the beta band has been paid attention on rather than tremor-related oscillations. And the role of cortical input was not considered before either. For example, Hu et al. [28] built a class of STN-GPe networks to explore beta oscillation conditions and found that the delays and coupling weights required for generating oscillations may decrease as the number of nuclei increases. Ahn et al. [31] found that Parkinsonian synchronized beta oscillations may be promoted by the simultaneous action of both cortical (or some other) and STN-GPe network mechanisms by a conductance-based models of the STN-GPe network and two types of input signals. Furthermore, Dovzhenok and Rubchinsky [34] showed that the strengthening of the basal ganglia-thalamo-cortical loop leads to tremor oscillations, while the weakening or disconnection of the loop suppresses them. However, they did not investigate the role of input from different pathways, and the model network included only single STN and GPe neurons.

In addition, previous researches mainly focused on the direct and indirect pathway [35], and the results by Leblois et al. [36] suggested that pathological synchronous oscillations in the BG can stem from a dynamical imbalance between the direct and hyperdirect pathways after dopamine depletion. In this paper, we explored the role of indirect and hyperdirect pathways on the synchrony and tremor-related oscillations in the BG network. Due to the complexity of multiple loops crossed with each other in the BG, the influences of these two pathways on shaping BG activity in PD state are analysed individually at first. Then, to investigate the mutual effect of them, cortical excitatory and striatal inhibitory inputs are modified simultaneously. Our study will allow for a deeper understanding of the mechanism of tremor-generating and may open a new treatment of the Parkinsonian tremor symptoms.

The content of this paper is organized as follows. The neuron model and the BG network are introduced in Section 2. Analyses of the effects of the indirect and hyperdirect pathways on synchrony and tremor-related oscillations are presented in Section 3. Finally, a brief conclusion is drawn in Section 4.

## 2. Models and Methods

### 2.1. The Basal Ganglia Network Model

**Figure 1:**
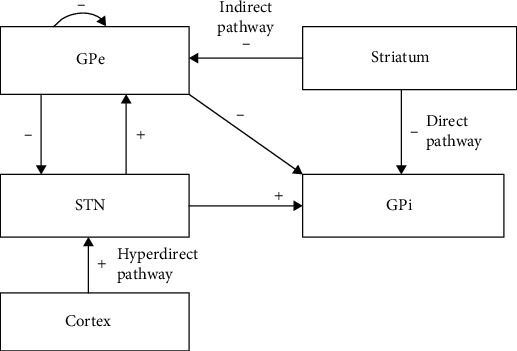
The structure and connections of the BG network model. Excitatory connections and inputs are represented with “+,” while inhibitory connections and inputs are represented with “-.”

The model used in this paper is based on So et al. [37], which was modified from the basal ganglia network model originally proposed by Rubin and Terman [38, 39]. In contrast to Rubin and Terman's model [38, 39], some changes were made to match more closely experimental data on neuron firing properties. And the BG network exhibited characteristics consistent with published experimental recordings, both on the level of single cells and on the network level [37]. The specific structure and connections of the network are illustrated in Figure 1. The network consists of four populations of 10 STN cells, 10 GPe cells, and 10 GPi cells each, connected to each other via synaptic connections. In the model, each STN cell receives inhibitory input from two GPe cells. Each GPe neuron receives inhibitory input from two other GPe cells and excitatory input from two STN neurons. Every GPi cell receives inhibitory input from two GPe neurons and excitatory input from two STN neurons. These projections are selected to match the physiological organization of synaptic connectivity within the basal ganglia more closely. And all connections are randomly chosen.

In addition to these detailed connections of internuclear or intranuclear, there are also some external current inputs from different pathways as depicted in Figure 1. All STN neurons receive excitatory input from the cortex through the hyperdirect pathway. Similarly, all GPe and GPi neurons receive striatal inputs via indirect and direct pathways.

### 2.2. The Single-Neuron Model in the BG Network

Models of STN, GPe, and GPi are represented by a single-compartment conductance-based model. The dynamics of the individual neuron for each part in the network is described by a set of differential equations as follows:

(1) $$\begin{array}{c} \left\{\begin{array}{c} C_{\text{m}}\frac{\text{d}V_{\text{STN}}}{\text{dt}}=-I_{\text{L}}-I_{\text{Na}}-I_{\text{K}}-I_{\text{T}}-I_{\text{Ca}}-I_{\text{ahp}}+I_{\text{syn}\_\text{STN}},\\ I_{\text{syn}\_\text{STN}}=-I_{\text{GPe}\to \text{STN}}-I_{\text{Cor}\to \text{STN}}+I_{\text{app}\_\text{STN}}, \end{array}\right. \end{array}$$

(2) $$\begin{array}{c} \left\{\begin{array}{c} C_{\text{m}}\frac{\text{d}V_{\text{GPe}}}{\text{dt}}=-I_{\text{L}}-I_{\text{Na}}-I_{\text{K}}-I_{\text{T}}-I_{\text{Ca}}-I_{\text{ahp}}+I_{\text{syn}\_\text{GPe}},\\ I_{\text{syn}\_\text{GPe}}=-I_{\text{GPe}\to \text{GPe}}+I_{\text{STN}\to \text{GPe}}+I_{\text{Str}\to \text{GPe}}+I_{\text{app}\_\text{GPe}}, \end{array}\right. \end{array}$$

(3) $$\begin{array}{c} \left\{\begin{array}{c} C_{\text{m}}\frac{\text{d}V_{\text{GPi}}}{\text{dt}}=-I_{\text{L}}-I_{\text{Na}}-I_{\text{K}}-I_{\text{T}}-I_{\text{Ca}}-I_{\text{ahp}}+I_{\text{syn}\_\text{GPi}},\\ I_{\text{syn}\_\text{GPi}}=-I_{\text{GPe}\to \text{GPi}}+I_{\text{STN}\to \text{GPi}}+I_{\text{Str}\to \text{GPi}}+I_{\text{app}\_\text{GPi}}, \end{array}\right. \end{array}$$

where *C*_m_ is the membrane capacitance, *I*_L_ is the leak current, *I*_K_ is the potassium current, *I*_Na_ is the sodium current, and *I*_T_ is the low-threshold T-type calcium current. *I*_Ca_ is the high-threshold calcium current, and *I*_ahp_ is the after-hyperpolarization potassium current.

As in Rubin and Terman [39], the synaptic current from the presynaptic cell *α* to the postsynaptic cell *β* is modeled as

(4) $$\begin{array}{c} I_{\alpha \to \beta }=g_{\alpha \to \beta }\left(V_{\beta }-E_{\alpha \to \beta }\right)\underset{j}{\sum }S_{\alpha }^{j}, \end{array}$$

where *g*_*α*→*β*_ is the maximal synaptic conductance between structure *α* and structure *β* and *E*_*α*→*β*_ is the reversal potential of the synaptic current. The summation is taken over all presynaptic neurons connected to the postsynaptic neuron.

For STN and GPi efferents, the synaptic variables are governed by a second-order alpha synapse to acquire an acute sense of synaptic effect as follows [37]:

(5) $$\begin{array}{c} \left\{\begin{array}{c} \frac{dS_{\alpha }^{j}}{dt}=z,\\ \frac{dz}{dt}=0.0234u\left(t\right)-0.4z-0.04S_{\alpha }^{j}. \end{array}\right. \end{array}$$

Here, *u*(*t*) = 1 if the presynaptic cell crosses a threshold of −10mV at time *t*, indicating the presence of an action potential in the presynaptic cell. Otherwise, *u*(*t*) = 0.

For all other synaptic connections, the synaptic variables are reserved as the following first-order differential equation [39]:

(6) $$\begin{array}{c} \left\{\begin{array}{c} \frac{dS_{\alpha }^{j}}{dt}=A_{\alpha }\left(1-S_{\alpha }^{j}\right)H_{\infty }\left(V_{\alpha }-\theta_{\alpha }\right)-B_{\alpha }S_{\alpha }^{j},\\ H_{\infty }\left(V\right)=\frac{1}{\exp\left(-\left(V-\theta_{g}^{H}\right)/\sigma_{g}^{H}\right)}, \end{array}\right. \end{array}$$

where *H*_∞_ is a smooth approximation of the Heaviside step function and *A*_*α*_, *B*_*α*_ control the synaptic time course. Details of the parameter values are given in [37].

### 2.3. Description of the Input from Different Pathways

There are three pathways within the basal ganglia: the direct pathway: Striatum⟶GPi, the indirect pathway:  Striatum⟶GPe⟶STN⟶GPi, and the hyperdirect pathway:  Cortex⟶STN⟶GPi [40]. In our simulations, the role of the direct and indirect striatal input applied to GPi and GPe is represented by the constant inhibitory current *I*_Str→GPi_ and *I*_Str→GPe_, respectively.

Furthermore, the primary role of the cortical neurons in the present study is to act as a source of excitatory input to STN transmitted through the hyperdirect pathway. In Kang and Lowery's computational study [40], the cortical input to STN is just modeled by a simple sine function. Here, to be more closer to reflect the biological properties, the excitation to STN from the cortex is modeled by an ungated channel with current referred to [40] as follows:

(7) $$\begin{array}{c} \left\{\begin{array}{c} I_{\text{Cor}\to \text{STN}}=g_{\text{ex}}\left(V_{\text{STN}}-V_{\text{cat}}\right),\\ g_{\text{ex}}=g_{\min}+0.01\sin\left(\frac{2\pi tf}{1000}\right), \end{array}\right. \end{array}$$

where *V*_STN_ is the membrane voltage of the STN neuron and *V*_cat_ is a cation reversal potential of 0 mV. In particular, *g*_ex_ is a sinusoidal conductance [41]. *g*_min_ can be tuned to produce experimentally observed firing rates, which can be viewed as the maximal synaptic conductance from cortex to the STN neuron. Additionally, *f* is the frequency of the cortical input in Hz.

### 2.4. Measurement of the Network Activity

Consistent with what is observed in humans with PD, the PD state in our model is characterized by synchronization and low-frequency oscillatory activity. To quantify the level of network synchrony and the proportion of low-frequency oscillations, as well as to identify how they are affected by network parameters in the BG network, several quantitative measures are introduced as follows:

#### 2.4.1. Synchrony Index

*(1) Voltage Synchrony Index*. The degree of synchrony in the network is calculated according to the method developed by [42–45]. The population averaged voltage *V*(*t*) is defined as *V*(*t*) = (*V*_1_(*t*)+⋯+*V*_*N*_(*t*))/*N*, where *N* is the number of cells, and then the voltage synchrony index SV is computed as follows:

(8) $$\begin{array}{c} \text{SV}=\frac{{\langle V{\left(t\right)}^{2}\rangle }_{t}-{\langle V\left(t\right)\rangle }_{t}^{2}}{1/N\sum_{i=1}^{N}\left({\langle V_{i}{\left(t\right)}^{2}\rangle }_{t}-{\langle V_{i}\left(t\right)\rangle }_{t}^{2}\right)}. \end{array}$$

The synchronization measure SV is normalized between 0 and 1, and the larger value of SV implies the higher degree of synchrony in the network.

*(2) Spike Synchrony Index*. Another synchrony measure, Spike synchrony (SS), was computed by modifying the voltage synchrony metric presented by Golomb and Rinzel [46], using counts of spikes binned into 5 ms bins. With *a*_*i*_(*t*) being defined as the binned spike count over time period for cell *i*, we computed the within cell variance *σ*_*i*_^2^ and population variance *σ*_pop_^2^ as follows:

(9) $$\begin{array}{c} \sigma_{i}^{2}={\langle a_{i}{\left(t\right)}^{2}\rangle }_{t}-{\langle a_{i}\left(t\right)\rangle }_{t}^{2},\sigma_{\text{pop}}^{2}={\langle a{\left(t\right)}^{2}\rangle }_{t}-{\langle a\left(t\right)\rangle }_{t}^{2}, \end{array}$$

where *a*(*t*) = (*a*_1_(*t*)+⋯+*a*_*N*_(*t*))/*N* for a population of *N* neurons and the brackets denote averaging over total simulation time. The synchrony measure SS was defined as:

(10) $$\begin{array}{c} \text{SS}=\frac{\sigma_{\text{pop}}^{2}}{1/N\sum_{i=1}^{N}\sigma_{\text{i}}^{2}}. \end{array}$$

Obviously, the larger the value of SS is, the higher the level of synchrony in the network becomes.

#### 2.4.2. Oscillation Index

It is well known that oscillations introduce peaks in the power spectral density of the population activity. Mean field potentials (MFPs) from a population were calculated to assess the degree of oscillations associated with tremor in the BG network. We estimated the spectrum *S*(*f*)_pop_ of the population activity by computing the Fast Fourier Transform of the MFP. Thus, the tremor oscillation index is defined as the relative power (referred to [47]) in the frequency band of 2-10 Hz as follows:

(11) $$\begin{array}{c} \text{OItremor}=\frac{\int_{2}^{10}S{\left(f\right)}_{\text{pop}}df}{\int_{0}^{30}S{\left(f\right)}_{\text{pop}}df}. \end{array}$$

Experimentally, Parkinsonian tremor may present with frequencies of 3-9 Hz according to [7], and 2-10 Hz oscillation chosen in our computation was sufficient to reflect the oscillatory firing associated with tremor in the BG network.

Besides, the peak power in the tremor frequency band is acquired by computing the maximal power within the range of 2-10 Hz, and it is denoted by peak tremor power as follows:

(12) $$\begin{array}{c} \text{Peak tremor power}=\underset{2\leq w\leq 10}{\max}\left\{P\left(w\right)\right\}, \end{array}$$

where *P*(*w*) is the power spectrum. And each power spectrum has been normalized with respect to the total power.

Similarly, the larger the value of tremor oscillation index OItremor is, the stronger of tremor-related oscillations in the network become.

### 2.5. Simulation Details

The state of the BG network can be changed by regulating the value of the external current applied to STN, GPe, and GPi. The switch from healthy condition to Parkinsonian condition in the model can be achieved by decreasing the constant bias currents *I*_app_*i*_(*i* ∈ { STN, GPe, GPi }), whose values for two different states are presented in Table 1. For the direct pathway of the BG, the value of *I*_Str→GPi_ is set to −2*μ*A/cm^2^.

Simulations were implemented in Matlab 2018a. The numerical method used was the forward Euler method with a time step of 0.01 ms. And the initial conditions are randomly chosen. Our numerical results were estimated after excluding the first 500 ms of initial network transients.

## 3. Results and Discussion

We mainly discussed the effects of indirect and hyperdirect pathway on synchrony and low frequency oscillations of the BG system. In the following simulations, the network initially remains PD state. Experimental studies have suggested that STN and GPe neurons play critical roles in the transmission of abnormal synchronized oscillatory activity throughout the entire BG in PD [48–50]. Therefore, the dynamics of these two nuclei are particularly concerned in the following discussions.

### 3.1. Effect of the Indirect Pathway on Synchrony and Tremor-Related Oscillations in the BG Network

To investigate the effect of the indirect pathway on the BG network, the hyperdirect pathway was blocked by removing cortical excitatory current input to STN, that is, the value of *I*_Cor→STN_ was tuned to 0 *μ*A/cm^2^.

Experimental studies have shown that the firing activity of Str-GPe projection greatly increased in PD state [51–55], possibly due to the altered synaptic activity in the striatum under the dopamine- (DA-) depleted condition [56, 57]. Specifically, striatal medium spiny neurons have been demonstrated to be more active after DA-depletion [58]. Therefore, the dynamics of the BG network under the influence of increased inhibitory input from the indirect striatum was studied in this part. In the simulation, we gradually increased the value of *I*_Str→GPe_.

#### 3.1.1. Indirect Striatal Activity Promotes Synchrony in the BG Network

**Figure 2:**
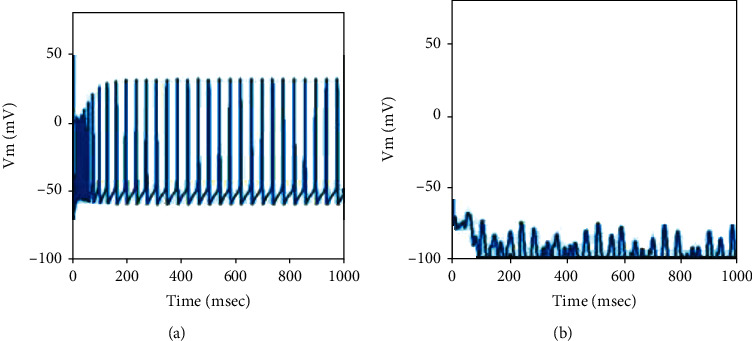
The firing of one randomly selected neuron in the BG network of (a) STN and (b) GPe cell when the value of *I*_Str→GPe_ is chosen as -16 *μ*A/cm^2^.

When the value of *I*_Str→GPe_ is less than -14 *μ*A/cm^2^, GPe cells cannot fire since the input from striatum is strong enough to restrain GPe nucleus, as shown in Figure 2(b) with *I*_Str→GPe_ being chosen as -16 *μ*A/cm^2^. Therefore, the value of *I*_Str→GPe_ is controlled in the range of -14 to 0 in what follows.

**Figure 3:**
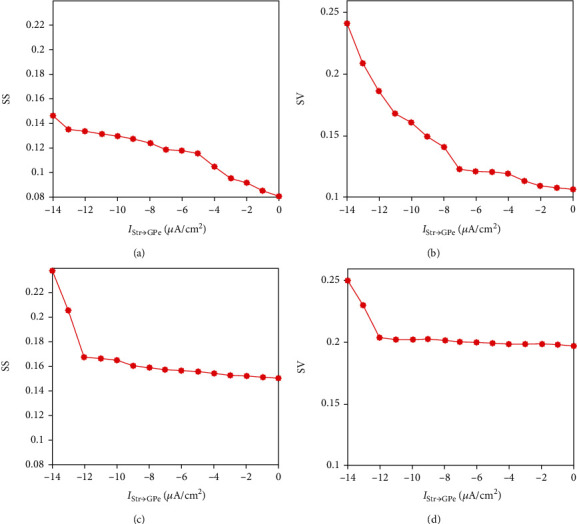
Evolutions of the synchrony index for different levels of striatal inhibition. (a) and (c) are spike synchrony index (SS) with the variation of *I*_Str→GPe_ for GPe and STN. (b) and (d) are voltage synchrony index (SV) with the variation of *I*_Str→GPe_ for GPe and STN.

Figure 3 shows how the level of synchrony in GPe and STN changes by modifying the striatal indirect current. Observe that synchrony for both populations was strengthened with the increase of *I*_Str→GPe_. Both the spike and voltage synchrony of GPe neurons changed near linearly as the input increases (as shown in Figures 3(a) and 3(b)). However, the evolution of SS and SV of STN slightly increased firstly and then sharply increased from *I*_Str→GPe_ = −12 *μ*A/cm^2^ as shown in Figures 3(c) and 3(d). This is possibly due to the fact that the striatal current directly acts on GPe and then on STN through the mutual coupling between STN and GPe. Furthermore, the network is sparsely connected and the current of the indirect pathway imposed on GPe needs to exceed a certain level to change the dynamical properties of STN [59].

**Figure 4:**
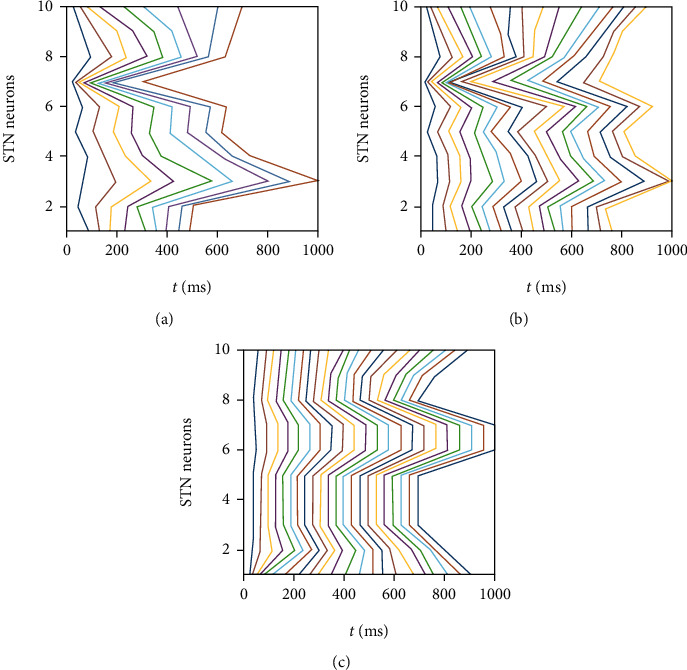
Connected spike rasters of 10 STN cells for three different values of *I*_Str→GPe_. (a) *I*_Str→GPe_ = −2 *μ*A/cm^2^. (b) *I*_Str→GPe_ = −8 *μ*A/cm^2^. (c) I_Str→GPe_ = −14 *μ*A/cm^2^.

To further illustrate the effect unambiguously, the spike timings for different STN neurons were connected as shown in Figure 4. Evidently, the straighter the line is, the stronger the synchrony becomes. In Figure 4(a), the activity for STN cells display irregular firing, which is only a little correlated when *I*_Str→GPe_ = −2 *μ*A/cm^2^. As the strength of the input increases to -8 *μ*A/cm^2^, there is rather more relevant firing among STN neurons, exhibiting higher correlated pattern as illustrated in Figure 4(b). Significantly, when the value of *I*_Str→GPe_ reaches -14 *μ*A/cm^2^, the lines in Figure 4(c) are almost flat, meaning that STN neurons achieve spike synchronization.

Our results suggest that increased striatal input from the indirect pathways can significantly promote synchrony in the BG network, which is in full accordance with the recent experimental evidences [60, 61].

#### 3.1.2. Indirect Striatal Activity Enhances Tremor-Related Oscillations in the BG Network

**Figure 5:**
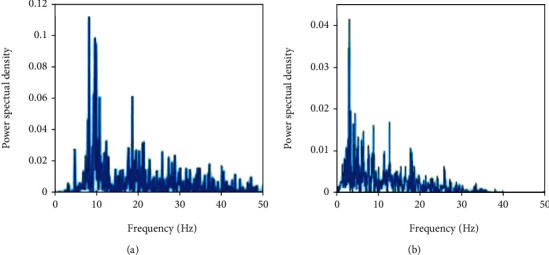
Power spectral density of (a) GPe and (b) STN populations under Parkinsonian state.

Under simulated dopamine depletion states, the oscillatory activities of both GPe and STN neurons at tremor and beta frequencies have been revealed in our model, as shown in Figure 5, which is in accordance with the physiological experiments [7, 9, 10]. The present available data strongly support that Parkinsonian tremor is related to the nigrostriatal dopaminergic deficit and other unexplained changes of firing characteristics within the basal ganglia loop [62]. In the BG network, the main afferent input comes through the striatum along the indirect pathway. Thus, we mainly focus on how the oscillations associated with tremor are affected by increased striatal activity after the reduction of dopaminergic terminals in the striatum.

**Figure 6:**
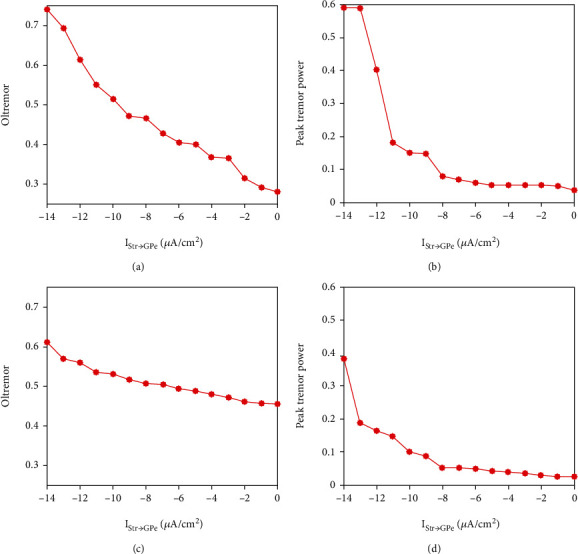
Evolution of tremor-related oscillations for different levels of striatal inhibition. (a) and (c) are oscillation index (OItremor) with the variation of *I*_Str→GPe_ for GPe and STN. (b) and (d) are the peak tremor power with the variation of *I*_Str→GPe_ for GPe and STN.

To identify the change of tremor-related oscillations in the model, two quantities, OItremor and peak tremor power, are calculated. Indeed, a progressive increase in inhibitory current to GPe promoted tremor-related oscillations in STN and GPe neurons. From Figures 6(b) and 6(d), we can see that the Peak tremor power increased moderately for both GPe and STN neurons firstly and then abruptly increased to more than 0.1 around *I*_Str→GPe_ = −11 *μ*A/cm^2^. Noteworthy, GPe neurons tend to yield more tremulous oscillatory activity than STN neurons (as shown in Figures 6(a) and 6(b)).

Anyhow, valuable results were obtained that increased striatal activities are sufficient to enhance tremor dynamics in the BG network. Compensatorily, the convincing arguments undertaken in [63] emphasized that the striatum might make important impacts in the development of the specific form of tremor. Our results provided additional verification for these findings.

### 3.2. Effect of Hyperdirect Pathway on Synchrony and Tremor-Related Oscillations in the BG Network

Similar to the previous investigation, to analyse the function of the hyperdirect pathway, the indirect pathway was blocked by removing striatal inhibitory current input to GPe, that is, *I*_Str→GPe_ = 0 *μ*A/cm^2^.

Electrophysiological studies have provided strong findings of a critical role for overactivity in the hyperdirect pathway contributing to the generation of abnormal synchronous activity following dopamine depletion [64, 65]. Analyses of functional connectivity in PD patients have further revealed that increased coupling between STN and cortical motor areas being associated with PD rigor and tremor symptoms [27, 66]. These indications suggest that the intensive cortex-BG connection may be pathophysiologically relevant and the dynamical properties of the BG network may be dependent on it. Based on the above results, the dynamics of the BG network under the influence of increased excitatory cortical input with different frequencies from the hyperdirect pathway is discussed in this section.

#### 3.2.1. Strong and High-Frequency Cortical Inputs Synchronized the BG Network

**Figure 7:**
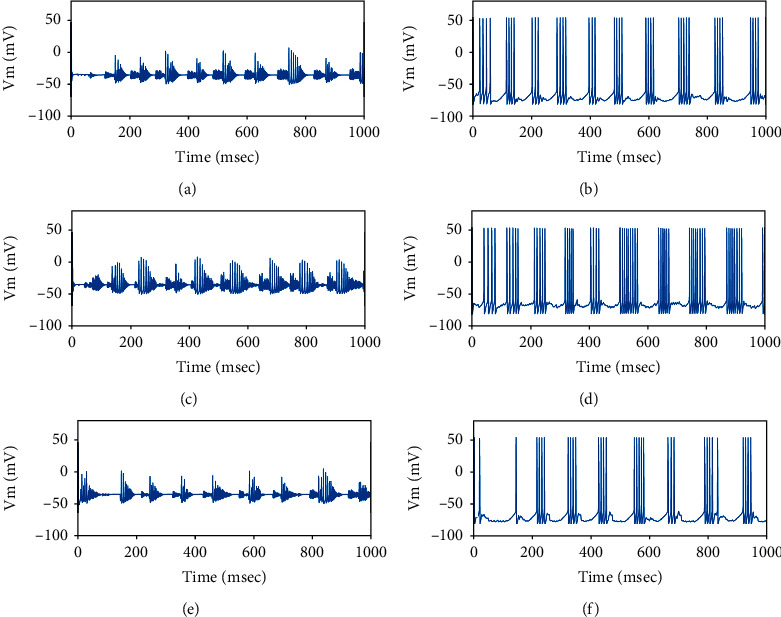
Membrane potentials of one randomly selected of STN and GPe neuron as the value of *g*_min_ is 1.4 uA/cm^2^ for three different frequency bands (*θ*, *β*, *γ*) of the cortical input. (a) and (b) are the case with *f* = 5 Hz, (c) and (d) are the case with *f* = 25 Hz, (e) and (f) are the case with *f* = 60 Hz.

**Figure 8:**
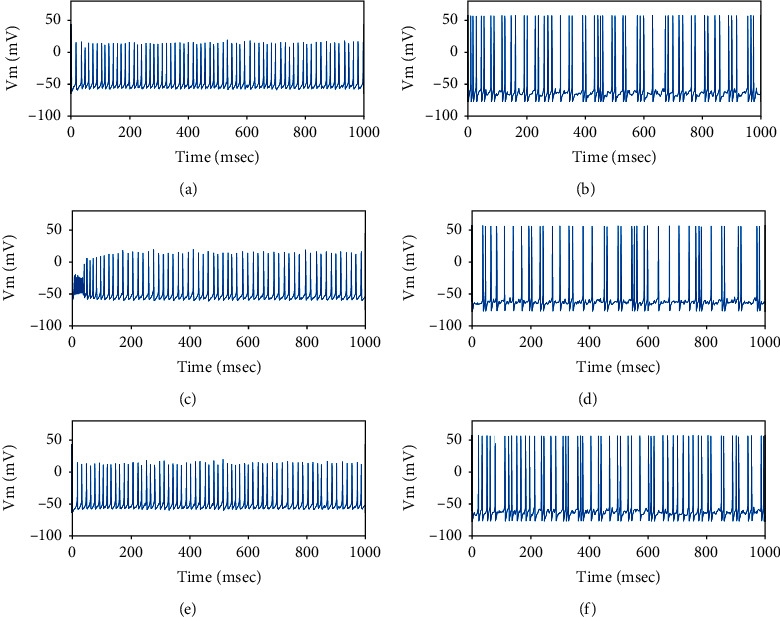
Membrane potentials of one randomly selected of STN and GPe neuron as the value of *g*_min_ is 0.8 *μ*A/cm^2^ for three different frequency bands (*θ*,  *β*,  *γ*) of the cortical input. (a) and (b) are the case with *f* = 5 Hz, (c) and (d) are the case with *f* = 25 Hz, (e) and (f) are the case with *f* = 60 Hz.

As mentioned in Section 2.3, the current transmitted by the hyperdirect pathway from the cortex to STN is modeled by (7). When the value of *g*_min_ is adjusted to exceed 1.2 *μ*A/cm^2^, the firing patterns of neurons in the basal ganglia are abnormal as shown in Figure 7. Thus, the value of *g*_min_ is controlled within 1.2 *μ*A/cm^2^  to ensure the normal patterns of the BG network. As can be seen from Figure 8, the firing of STN and GPe is normal when *g*_min_ = 0.8 *μ*A/cm^2^.

**Figure 9:**
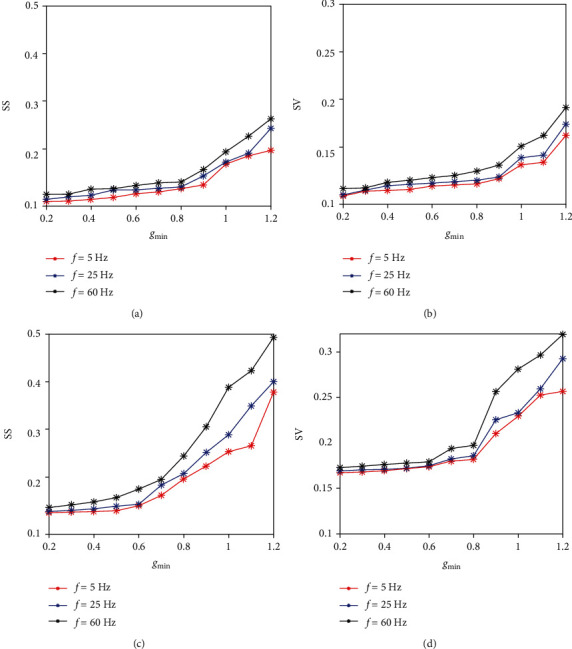
Evolutions of the synchrony index for different strengths of the synaptic gain from the cortex to STN in different frequency bands (*θ* = 5 Hz,  *β* = 25 Hz,  *γ* = 60 Hz). (a) and (c) are the spike synchrony index (SS) with the variation of *g*_min_ for GPe and STN. (b) and (d) are the voltage synchrony index (SV) with the variation of *g*_min_ for GPe and STN.

Figure 9 illustrates how the degree of synchrony in BG depends on the input from the cortex to STN for three specific frequencies in different frequency bands (*θ*, *β*, *γ*). Note that the BG network becomes more synchronized when the intensity of the gain between the cortex and STN is increased. The degrees of synchronization for STN and GPe neurons are dramatically strengthened when *g*_min_ is increased to 1.0 *μ*A/c*m*^2^. When an even more powerful input from the cortex to STN is applied, the abnormal synchrony is extremely enhanced in both nuclei. These results indicate that a pathologic cortical-STN coupling might be a crucial mediator in the pathophysiology of PD, which is consistent with experimental analysis [27]. Furthermore, as can be seen from Figures 9(a)–9(c), the value of SS for STN population is greater than that for GPe population for all three different frequencies. This means that STN is a key node in the basal ganglia of PD patients. In addition, the values of SS and SV for STN and GPe cells are larger when the frequency of cortical input is as high as in the gamma band, which shows that the synchronization of BG network is strongly enhanced with high-frequency input from cortex. These results quite support that interference of the motor cortex could lead to widespread restoration of movement dysfunction, and it could be a potentially therapeutic approach.

#### 3.2.2. Strong and High-Frequency Cortical Input Facilitates Tremor Oscillations in the BG Network

Cortical tremor-related oscillations has been found by magnetoencephalography [16] and electroencephalography [17] in patients with PD. Therefore, we predict that those observed low-frequency tremor oscillations in the BG network in the experiments may come from the input from the cortex. And a deeper explanation of this mechanism is presented in the following.

**Figure 10:**
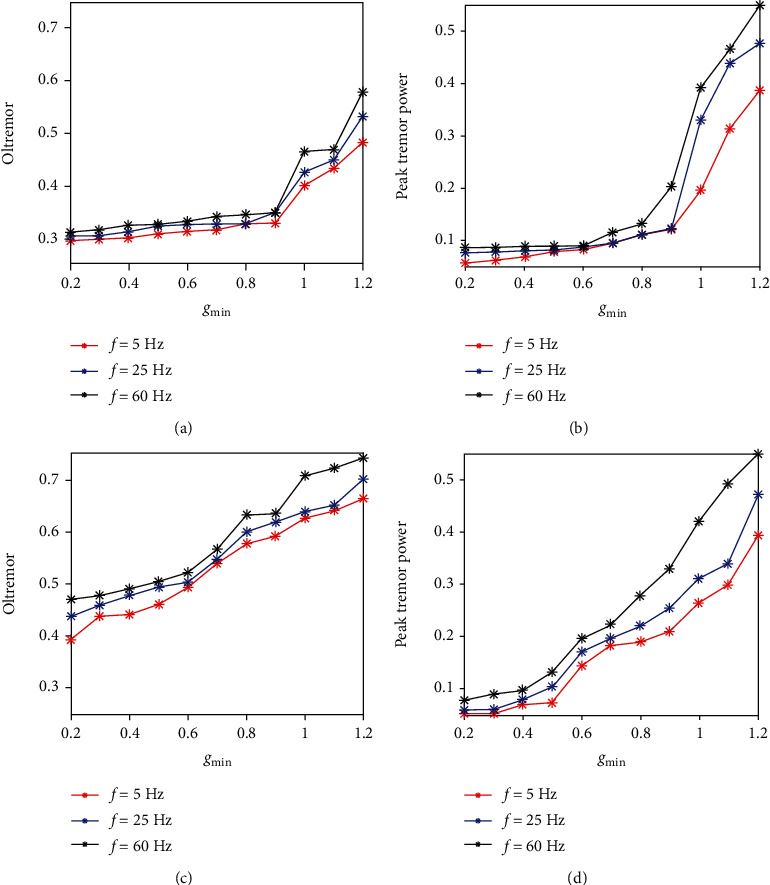
Evolutions of tremor-related oscillations for different strengths of the synaptic gain from the Cortex to STN in different frequency bands (*θ* = 5 Hz,  *β* = 25 Hz,  *γ* = 60 Hz) of cortical input. (a) and (c) are the Oscillation index (OItremor) with the variation of *g*_min_ for GPe and STN. (b) and (d) are the peak tremor power with the variation of *g*_min_ for GPe and STN.

The role of increased input from the cortex to STN for three frequencies in different frequency bands (*θ*, *β*, *γ*) on tremor-related oscillations in BG network is presented in Figure 10. It is obvious that the tremor-related oscillations in STN and GPe populations are strengthened as the value of *g*_min_ is increased. And tremor-related oscillations in GPe neurons increase dramatically when the value of *g*_min_ increases from 0.9 *μ*A/cm^2^ as shown in Figures 10(a) and 10(b). This means that oscillations in tremor frequencies in GPe population can be explosively increased when STN cells become sufficiently active. In contrast, as Figures 10(c) and 10(d) shows, tremor-related oscillations in STN population increased linearly for all three frequencies of cortical inputs. These results match well with earlier experimental reports [67] that cortical activity can lead to low-frequency oscillatory activity in the STN-GPe network in the dopamine-depleted state.

In addition, tremor-related oscillations in both STN and GPe populations are stronger with high-frequency inputs than low-frequency ones. This finding suggests that high-frequency gamma activity in the cortex is interacting with slow oscillations in the STN-GPe network, which may lead to the aggravation of tremor-related oscillations. Correspondingly, various strands of experimental analysis [68, 69] have disclosed the important role of cross-frequency interactions across multiple neural structures.

### 3.3. The Mutual Effect of Indirect and Hyperdirect Pathways on Synchrony and Tremor-Related Oscillations in BG Network

The individual influences of indirect and hyperdirect pathways on the basal ganglia following dopamine depletion were revealed in the previous sections. To examine the mutual effect of them, the cortical excitatory current to STN and striatal inhibitory current to GPe are introduced simultaneously. For simplicity, the SS index and peak tremor power index mentioned above are selected to evaluate the degree of synchronous activity and tremor-related oscillations in the BG network.

**Figure 11:**
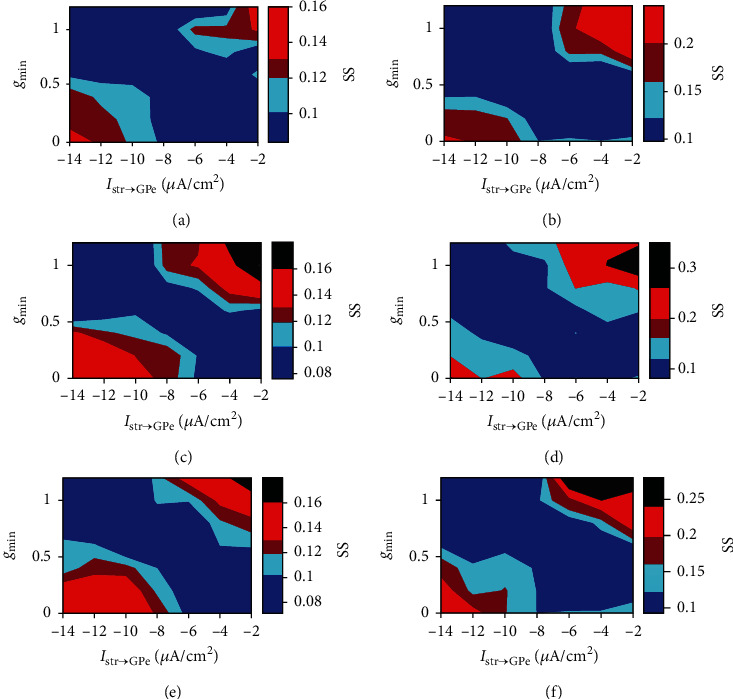
Transitions of synchronous behavior in dependence of *g*_min_ and *I*_Str→GPe_ for different frequency bands of cortical input (*θ* = 5 Hz,  *β* = 25 Hz,  *γ* = 60 Hz). Colors in the *g*_min_-*I*_Str→GPe_ plane represent the value of spike synchrony index (SS). (a) and (b) are the case with *f* = 5 Hz for GPe and STN. (c) and (d) are the case with *f* = 25 Hz for GPe and STN. (e) and (f) are the case with *f* = 60 Hz for GPe and STN.

**Figure 12:**
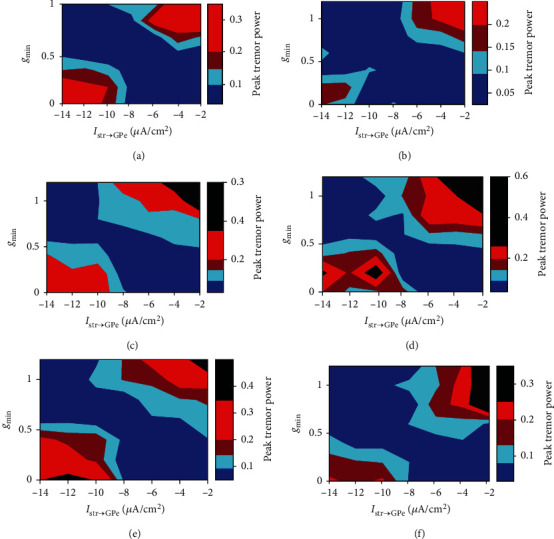
Evolutions of tremulous behavior in dependence of *g*_min_ and *I*_Str→GPe_ for different frequency bands of cortical input (*θ* = 5 Hz,  *β* = 25 Hz,  *γ* = 60 Hz). Colors in the *g*_min_-*I*_Str→GPe_ plane represent the value of Peak tremor power. (a) and (b) are the case with *f* = 5 Hz for GPe and STN. (c) and (d) are the case with *f* = 25 Hz for GPe and STN. (e) and (f) are the case with *f* = 60 Hz for GPe and STN.

Figures 11 and 12 show how the level of neuronal synchrony and tremor-related oscillations in STN and GPe change under three different frequencies of cortical input. It is obvious that the degree of synchronization and tremor-related oscillations for STN and GPe populations are much stronger in the regions of higher *g*_min_ and lower *I*_Str→GPe_ (upper right) or lower *g*_min_ and higher *I*_Str→GPe_ (bottom left). This means that the network would become highly synchronized and tremulous if the strength between striatal inhibition to GPe and cortical excitation to STN is greatly imbalanced. And these abnormal dynamics are suppressed by tuning the network into less synchronized and less tremulous oscillations as these two parameters are modulated to be relatively balanced.

In addition, compared to low theta input from cortex, the areas of synchronization and tremor-related oscillations are increased for cortical beta or gamma inputs (as can be seen in Figures 11 and 12). And in this situation, the degree of synchrony and oscillatory activity in the tremor frequency are much stronger at higher *g*_min_ and lower *I*_Str→GPe_ regions.

These results suggest that the balance between striatal inhibition from the indirect pathway and cortical excitation from the hyperdirect pathway is significant for maintaining the normal dynamics of the basal ganglia. It provides an effective way to prevent Parkinson's disease by measuring the strength of currents from these two paths and intervening with medication. Furthermore, the symptoms of tremor may be alleviated by controlling the frequency of the current from the cortex to STN within the theta band in the clinical treatment.

## 4. Conclusion

In this paper, we studied how the synchronization and tremor-related oscillations in the BG network are affected by the indirect and hyperdirect pathways after dopamine depletion. A modified Hodgkin-Huxley type model of the BG network was used to mimic the four structures of BG. To allow a more complete framework to investigate the role of these two paths, a representation of cortical inputs and the inhibitory striatal input are introduced in this model. The extended model presented here can provide more comprehensive insight into the possible origins of tremor-related dynamics than previous models, which have considered only the role of the single BG network in the generation of Parkinson.

Here, due to the complex nature of multiple loops in the basal ganglia, the influences of these two pathways are analysed individually at first. To explore the function of the indirect pathway, the inhibitory current from the striatum to GPe was adjusted. And our results demonstrated that increased inhibitory activity from the striatum to GPe can be effective to enhance synchronized activity and tremor-related oscillations in the basal ganglia. And Kumar et al. [47] showed that the strength of inhibitory inputs from striatum to GPe is a key parameter controlling beta oscillations in the BG by a large-scale spiking neural network model of the STN-GPe loop. To analyse the role of the hyperdirect pathway, the applied cortical current was regulated. And our results suggested that strong and high gamma cortical input can significantly synchronize the BG network and enhance the tremor-related oscillations. This indicates that the interaction of high-frequency gamma input from the cortex and low frequency oscillations in the STN-GPe network can lead to the presence of tremor-related oscillations. This may explain the significant role of cross-frequency interactions between neural structures. Meanwhile, Shouno et al. [70] showed that cortical excitatory input to the STN can influence pathological STN oscillations by a spiking neuron model of the STN-GPe circuit, which predicts the important role of cortical inputs to the STN.

Then, to obtain the mutual effect of these two pathways, the cortical excitatory and striatal inhibitory currents were modified simultaneously. Our results suggested that the network would be in a highly synchronized and tremulous state if the role between the indirect pathway and the hyperdirect pathway is greatly imbalanced. Conversely, the network may move to a less synchronized and less tremulous state when the strengths between these two paths are relatively balanced. These findings support that an imbalanced role of these two pathways in the basal ganglia is important for the generation of abnormal activities. Holgado et al. [71] found that the excitatory input from the cortex to STN needs to be high relative to the inhibition from striatum to GPe is a necessary condition for generation of oscillations, which can support our results in a way.

Taken together the above findings, it can be concluded that an imbalance among the pathways may be the origin of the synchrony and tremor-related oscillations in the BG network. And our study may provide a novel treatment in the cortex and striatum to alleviate the symptom of tremor in Parkinson's disease.

## Figures and Tables

**Table 1:** Model parameters under healthy and Parkinsonian conditions.

Conditions	*I*_app_STN_ (*μ*A/cm^2^)	*I*_app_GPe_ (*μ*A/cm^2^)	*I*_app_GPi_ (*μ*A/cm^2^)
Healthy	33	20	23
Parkinsonian	23	7	17

## Data Availability

The data is available upon request.
